# Delayed diagnosis of angioimmunoblast T-cell lymphoma presenting with immunoglobulin a vasculitis

**DOI:** 10.1080/0886022X.2024.2416935

**Published:** 2024-10-28

**Authors:** Lu Xu, Huihui Dong, Dandan Xue, Yiming Zhang, Xinjian Li

**Affiliations:** a Department of Hemopurification, Affiliated Hospital of Jining Medical College, Jining, China; b Department of Nephrology, Yanzhou District People’s Hospital, Jining City, China; c Department of Nephrology, Affiliated Hospital of Jining Medical College, Jining, China

**Keywords:** IgA vasculitis, angioimmunoblastic T-cell lymphoma, Henoch–Schonlein purpura, delayed diagnosis, case report

## Abstract

Although vasculitis and renal involvement might be associated with malignancy, they are rarely associated with lymphoproliferative diseases. We observed a case of immunoglobulin A vasculitis associated with segmental and focal glomerulonephritis in a patient with Angioimmunoblastic T-cell lymphoma. The most interesting aspect of this case is that this patient’s main initial manifestations were skin rash and fever. No significant lymph node enlargement was found at the time. Routine urine examination found positive urine protein, and renal puncture biopsy indicated purpura nephritis. More than one year later, the patient was admitted to the Department of Hematology of our hospital again after finding a mass in the neck. After that, the patient was diagnosed with Angioimmunoblastic T-cell lymphoma by lymph node biopsy. To our great surprise, successful treatment of the underlying malignancy was associated with the remission of glomerulonephritis and cutaneous vasculitis.

Angioimmunoblastic T-cell lymphoma (AITL) is an infrequent hematological malignancy with variable and often atypical presentations. The presence of dysproteinemia, autoantibodies, and systemic involvement in AITL have often led to a delay in diagnosis or even misdiagnosis in clinical practice. We report a case of AITL that primarily presented with skin purpura associated with immunoglobulin A (IgA) vasculitis.

A 48-year-old male was transferred to our department on 3 March 2016 with a complaint of general rash for two months. When skin purpura initially appeared, the patient was treated in a local hospital with ‘compound rutin tablets, vitamin B6, and prednisone acetate.’ The skin purpura gradually disappeared. However, when systemic rash appeared and persisted for two days, the patient arrived at our hospital to diagnose the disease. An examination revealed unchanged superficial lymph nodes, absent eyelid, lower limb edema, and a heart rate of 80 beats/min. The abdomen, buttocks, and lower limbs were scattered with purplish red, stasis-point ecchymosis rash that did not fade under pressure, with local fusion. The laboratory findings included white blood cells, 5.24 (3.5–9.5) × 10^9^/L; hemoglobin, 139 g/L (115–150 g/L); platelets, 273 (125–350) × 10^9^/L. Lymphocytes constituted 37.8% (20–50%) of the white blood cells, with a concentration of 1.98 (1.1–3.2) × 10^9^/L. Urinalysis found protein 1+, occult blood 2+, and urine protein quantification in 24 h was 1.89 g. Urine protein electrophoresis indicated glomerular proteinuria, creatinine 68.7 µmol/L, and urea nitrogen 2.9 mmol/L. No abnormality was found in tumor markers, immune indexes, or immunofixation electrophoresis. The patient underwent renal biopsy a month after presenting at our department, consistent with purpura nephritis of focal segment necrosis with mild mesangial proliferative changes. This biopsy supported a diagnosis of IgA vasculitis for which he was started on methylprednisolone. In July 2016, the patient developed fever with a maximum body temperature of 38 °C, accompanied by dizziness, headache, nasal congestion, runny nose, cough, expectoration, and itching skin scattered with erythema and papules. Chest computed tomography (CT) examination showed inflammation in the right upper lung lobe and scattered small lymph nodes in the mediastinum. The laboratory findings were: red blood cell, 4.05 × 10^12^/L; hemoglobin, 124 g/L; white blood cells, 22.16 × 10^9^/L; 86.7% neutrophils; platelets, 258 × 10^9^/L; C-reactive protein, 41.36 mg/L. Urine protein in 24 h was 0.85 g. The patient was treated with cefoperazone and sulbactam for seven days, and was then discharged as the body temperature dropped to normal level and the general condition improved.

In February 2017, the patient was admitted to hospital again for fever, rash, and itching. The skin all over the body was scattered with erythema, papules, and wind masses, but no blisters. The patient underwent another chest CT examination, demonstrating chronic inflammation of the upper right lung lobe and multiple enlarged mediastinal lymph nodes. The patient refused to undergo color Doppler ultrasonography of the superficial lymph nodes with lymph node biopsy if indicated.

In July 2017, the patient was transferred to the hematology department with complaint of a neck tumor discovered a month earlier. Subsequently, the patient underwent superficial lymph node color Doppler examination, which revealed multiple hypoechoic nodules on both sides of the neck. The largest nodules were about 1.8 × 0.7 × 1.4 cm on the right, and 1.7 × 0.7 × 1.1 cm on the left. The nodules had obscure borders, uneven internal echo, with internal detectable rich blood flow signal. Multiple hypoechoic nodules were also seen in the bilateral groin, with the largest on the left of about 3.2 × 1.3 × 2.3 cm. Chest CT demonstrated multiple enlarged lymph nodes in the mediastinum, bilateral hilum, axillary, and subclavian fossa, right epicenteric angle, hepatogastric space, and retroperitoneum. Right inguinal resection was performed the following day. Histopathology indicated angioimmunoblastic T-cell lymphoma(AITL). Effective and durable disease control of AITL depends on combined chemotherapy and/or autologous stem cell transplantation.

Following internal discussion, the patient was given another course of GDP(gemcitabine, dexamethasone, cisplatin). One month after chemotherapy, the patient was negative for urinary protein (0.10 g/24 h). After using the GDP regime, the patient ‘s condition was controlled, no rash appeared, and the lymph nodes were also reduced. The patient ‘s urine protein continued to be negative during subsequent chemotherapy. The patient eventually died of pulmonary aspergillosis in August 2022.

This was a typical case of non-Hodgkin lymphoma misdiagnosed as allergic purpura nephritis for over a year. A time lag in the diagnosis of AITL is not uncommon as the early symptoms may be multifarious and nonspecific in nature [1]. AITL is a rare tumor that accounts for 1% of all lymphoma cases. It is characterized by the loss of the lymphoid architecture, presence of pleomorphic cellular infiltrates, and proliferation of microvasculature in the lymph nodes. Patients usually present with fever, generalized lymphadenopathy, skin rash, polyclonal hypergammaglobulinemia, Coombs-positive anemia, thrombocytopenia, and hypocomplementemia [2]. Renal involvement is rare in patients with AITL. However, some cases involving proteinuria, nephrotic syndrome [3], acute renal failure [4], and membranous nephropathy [5] have been reported.

This patient was misdiagnosed for more than one year. During the course of the disease, there were two fevers, and the inflammatory index was high, so we all classified it as caused by infection. In fact, the patient’s second fever and chest CT suggested that there were multiple mediastinal lymph nodes enlargement. Until July 2017, the patient came to the hospital with a neck mass, and underwent mass resection. The pathological results indicated vascular immunoblastic T-cell lymphoma. The proteinuria disappeared after chemotherapy with a GDP regimen (gemcitabine, dexamethasone, cisplatin). We therefore assumed that the patient’s IgA vasculitis was associated with the AITL.

IgA vasculitis was previously known as Henoch-Schonlein purpura. It is a small-vessel vasculitis that typically involves the skin, gut, and kidney and is characterized by IgA deposition in affected blood vessels [6]. It has usually been regarded an immune complex disease; however, there has been increasing interest in the concept that mesangial IgA deposition in lgA vasculitis might occur through features of the IgA molecule independently of classical immune complex formation, particularly through altered glycosylation of the IgA1 molecule [7]. Tumor antigens might lead to ­systemic T-cell-driven B-cell production of lgA1, consistent with the presence of IgAl in the glomerulus in lgA ­vasculitis [8].

Therefore, future clinical work in older adult patients diagnosed with IgA vasculitis should exclude the possibility of a tumor, thereby reducing the misdiagnosis time and achieving good treatment effect. Clinicians are advised to consider multi-faceted lymphoma in various clinical scenarios of auto-immune diseases.
